# Yoga an integrated mind body intervention for improvement in quality of life in individuals with Alzheimer’s disease and their caregivers

**DOI:** 10.3389/fragi.2025.1449485

**Published:** 2025-03-21

**Authors:** Meenakshi Kaushik, Anjali Yadav, Ashishdatt Upadhyay, Anu Gupta, Prabhakar Tiwari, Manjari Tripathi, Rima Dada

**Affiliations:** ^1^ Lab. for Molecular Reproduction and Genetics, Department of Anatomy, All India Institute of Medical Sciences, New Delhi, India; ^2^ Department of Clinical Research Unit, All India Institute of Medical Sciences, New Delhi, India; ^3^ Department of Neurology, All India Institute of Medical Sciences, New Delhi, India

**Keywords:** Alzheimer’s disease, quality of life, yoga, meditation, GDS &MoCA scales, caregiver burden

## Abstract

**Background and objective:**

Alzheimer’s disease (AD) presents profound challenges, significantly impairing quality of life (QOL) for patients and increasing the burden on caregivers. This study aims to investigate the effectiveness of a tailored 12-week yoga intervention in improving the quality of life for individuals with mild to moderate AD and reducing caregiver burden (CB).

**Methods:**

This is yoga interventional study with healthy controls enrolled 30 participants (18 males, 12 females) diagnosed with mild to moderate AD by an expert neurologist. Participants were aged 60 years or older and were recruited from an old age home. A 12-week yoga program, including specific postures (asanas), pranayama (breathing exercises), and meditation, was conducted for 1 hour daily, 6 days a week. Neurocognitive assessments were performed pre- and post-intervention using the Geriatric Depression Scale (GDS), Montreal Cognitive Assessment (MoCA), and Caregiver Burden (CB) Scale. MoCA scores were analyzed across specific domains, including language, memory, attention, visuospatial ability, delayed recall, abstraction, and orientation.

**Results:**

The intervention led to significant improvements in quality-of-life measures. GDS scores decreased from 8.36 ± 2.79 to 5.13 ± 3.07 (P < 0.01; 95% CI: −3.98 to −2.31), while MoCA total scores improved from 18.23 ± 4.90 to 21.10 ± 5.09 (P < 0.01; 95% CI: 2.17–3.89). Domain-specific MoCA scores also showed significant enhancements, particularly in language, attention, and delayed recall. Caregiver burden, measured using the CB Scale, demonstrated notable reductions following the intervention (P < 0.01; 95% CI: −2.54 to −1.23).

**Conclusion:**

This study underscores the significant improvements in depression and cognitive function, and overall quality of life in individuals with mild to moderate AD. Additionally, the intervention alleviated caregiver burden, highlighting its potential as an effective mind-body approach for AD management.

## Introduction

Alzheimer’s disease (AD) is a progressive, irreversible neurodegenerative disorder that affects over 50 million individuals worldwide. It gradually impairs cognitive and behavioral abilities, including memory, language, and reasoning, with symptoms worsening over time. AD is categorized into stages such as mild, preclinical, and dementia, based on the level of cognitive impairment (CI) (Kumar et al., 2024). The exact cause of AD is multifactorial, involving age—its most significant risk factor—alongside genetics, lifestyle factors (e.g., physical activity, diet, alcohol consumption, and smoking), education, and environmental influences (Alzheimer’s disease facts and figures, 2024). Global demographic shifts, as projected by the World Health Organization (WHO), underscore the growing prevalence of age-related conditions like AD, with the population aged 65 and older rising to 10% by 2023 and expected to reach 22% by 2050 (“2023 Alzheimer’s Disease Facts and Figures,” 2023). The interplay between genetic predispositions, such as the APOE4 allele, and modifiable lifestyle factors is critical in understanding AD progression. The APOE4 allele accelerates amyloid-beta (Aβ) accumulation and tau pathology (Cicognola et al., 2025), while lifestyle interventions like regular physical activity and adherence to a Mediterranean diet mitigate these effects by reducing neuroinflammation and enhancing neuroplasticity. For instance, physical exercise lowers amyloid burden in APOE4 carriers, and diets rich in antioxidants and omega-3 fatty acids improve cognitive resilience (Norwitz et al., 2021). Despite advances in understanding AD pathology, the condition remains characterized by three primary features: tau phosphorylation and tangles, activation of inflammatory pathways, and (Aβ) aggregation (Liu et al., 2024). The World Health Organization (WHO) defines quality of life (QOL) as an individual’s perception of their position in life, taking into account the cultural and value; systems in which they live, along with their goals, expectations, standards, and concerns. This comprehensive definition includes six key dimensions: (1) physical health, (2) psychological wellbeing, (3) level of independence, (4) social relationships, (5) environmental factors, and (6) spiritual beliefs. These dimensions collectively shape an individual’s overall sense of wellbeing and life satisfaction (Tahami Monfared et al., 2024). Given that QOLis a subjective concept, there is much debate on who should complete an assessment tool designed to gauge the QOL of those suffering from AD. The type of respondent in the literature is correlated with the severity of the dementia; assessment tools for patients with mild to moderate cognitive impairment use patient or caregiver reports, or both; in severe cases, the caregiver provides the only information (Gumikiriza-Onoria et al., 2024). The Geriatric Depression Scale (GDS), initially developed by Yesavage et al., has been extensively tested and utilised with the elderly population, despite the fact that there are other tools available to measure depression. The GDS Long Form is a short, thirty-item survey that asks participants to rate their feelings from the previous week by selecting yes or no. In 1986, a set of 15 questions known as the Short Form GDS was created. The GDS Short Form was created by condensing questions from the Long Form that, in validation trials, showed the strongest connection with depressed symptoms (Grill et al., 2024). As a screening tool to identify people with MCI, the MoCA was created in 2005 (Nasreddine et al., 2005). Even though the MMSE has been widely used since its creation, it is not sensitive enough to identify mild cognitive impairment (MCI) in people who are at risk of developing AD. The MoCA was designed to focus on the impairment domains that are most frequently seen in MCI patients. It also consistently exhibits substantially higher sensitivity in identifying MCI and AD (Malek-Ahmadi and Nikkhahmanesh, 2024). As AD progresses, patients’ independence declines, necessitating increased caregiver involvement, which can lead to heightened stress and anxiety. Caregivers often experience burnout when they take on too much responsibility, leading to negative emotional and physical consequences (Gómez-Gallego and Gómez-Gallego, 2021). Research has shown that factors such as caregiver personality, the patient’s level of functional impairment, environmental barriers, and the availability of support systems contribute significantly to caregiver burden (CB) (Pudelewicz et al., 2019; Monteiro et al., 2024). These factors influence the caregiver’s QOL and their ability to manage caregiving tasks effectively. Additionally, CB can be assessed using established measures such as the Caregiver Burden Inventory and the Zarit Burden Interview, which evaluate the emotional, physical, and social strain of caregiving.

Yoga, an ancient mind-body discipline, has emerged as a promising approach for enhancing cognitive and emotional health. Studies have demonstrated its ability to improve mental and physical wellbeing, reduce depression severity, and delay or prevent the onset of AD in older adults (Tolahunase et al., 2018a). Seniors experiencing age-related cognitive decline can benefit yoga-therapy that increase their cognitive abilities and is totally safe and can be practiced with caregivers and can also benefit not only AD individuals but also caretakers. It has been documented that yoga has the potential to lessen the primary risk factors of AD in late life, such as stress, anxiety, and depression, along with sleep disturbances. Studies on the impact of yoga in managing complex lifestyle diseases and major depressive disorder (MDD) have demonstrated that yoga enhances the secretion of key neurochemicals, including Brain-Derived Neurotrophic Factor (BDNF), serotonin, and melatonin. These molecules play a crucial role in promoting neuroplasticity, which improves the brain’s ability to adapt and reorganize. This neuroplastic enhancement is associated with significant reductions in stress, anxiety, and depression severity (Tolahunase et al., 2018a; Tolahunase et al., 2018b; Dada et al., 2022; Gautam et al., 2019).

Furthermore, yoga has been shown to improve cognitive function in individuals with MCI and delay its progression to AD (Ibrahim et al., 2022). Research from our laboratory corroborates these findings, highlighting the role of yoga in reducing systemic inflammation and promoting factors that enhance neuroplasticity, such as BDNF. These studies span a range of conditions, including glaucoma, depression, rheumatoid arthritis, and male infertility, further underscoring yoga’s broad therapeutic potential (Tolahunase et al., 2018b; Gautam et al., 2019; Bisht et al., 2017). Together, this body of evidence establishes yoga as a holistic intervention for improving both cognitive and emotional health via pathways mediated by BDNF, serotonin, and melatonin. Given India’s rapidly aging population and the increasing burden of AD, the relevance of yoga as a culturally grounded, non-pharmacological intervention is particularly strong. This study is the first to investigate yoga’s impact on cognitive decline in an Indian cohort with MCI and AD. The findings underscore the potential of yoga as an early intervention strategy to slow or prevent cognitive deterioration, addressing critical gaps in AD management and research.

## Methods

### Study design and subjects’ recruitments

**FIGURE 1:**
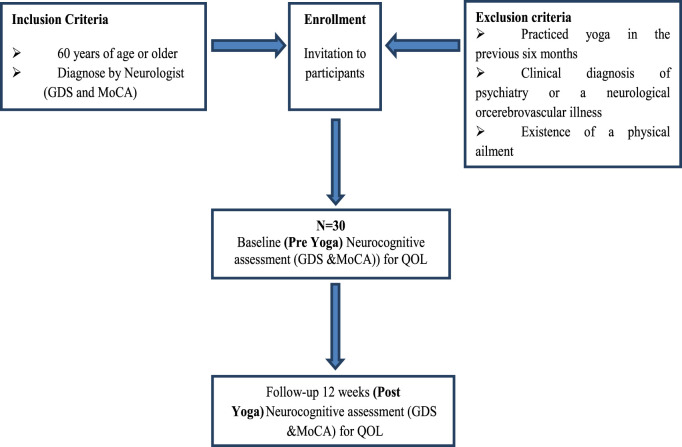
Schematic Representation of the study flow. The flowchart represents the participant selection and study design for a neurocognitive assessment of a yoga intervention. Participants are included if they are 60 years or older or have been diagnosed with a condition by a medical professional. Exclusion criteria include prior yoga practice within the last 6 months, a clinical diagnosis of psychiatric, neurological, or cerebrovascular illness, or the presence of a physical ailment. Eligible participants receive an invitation to enroll in the study. A total of 30 participants (N = 30) undergo a baseline (pre-yoga) neurocognitive assessment using the Geriatric Depression Scale (GDS) and the Montreal Cognitive Assessment (MoCA). Following a 12-week yoga intervention, participants undergo a post-yoga neurocognitive assessment using the same tools, along with an evaluation of Quality of Life (QOL).

This study employed a case-control yoga-based intervention with a 12-week follow-up period. Prior to the study, ethical approval was obtained from IEC of AIIMS, New Delhi, India and patients were enrolled after providing informed consent. A total of 30 participants were recruited from an old-age home, and their diagnoses were performed in the Department of Neurology by an expert neurologist. The yoga intervention sessions were conducted in the Department of Anatomy by a trained yoga therapist. A detailed outline of the study flow diagram is presented in Figure 1. Participants with MCI and AD, aged 60 years or older, were enrolled, along with their caregivers, based on the following eligibility criteria (Kilpatrick et al., 2020).

### Inclusion criteria

1. Participants attending the Department of Neurology, AIIMS, New Delhi.
2. Age 60 years or older.
3. Diagnosis of MCI or AD confirmed by a neurologist based on patient-reported memory problems verified by an informant (formal or informal caregiver) and a MoCA score of less than 21 (MoCA <21).
4. Clinical diagnosis of AD as per the NINCDS-ADRDA workgroup criteria and DSM IV–R.
5. Completion of medical and neurological evaluations, routine blood tests, and brain MRI.

### Exclusion criteria

1. Participants who had practiced yoga in the previous 6 months.
2. Participants with a clinical diagnosis of neuropsychiatric disorders, neurological illnesses, or cerebrovascular conditions.
3. Patients with other complications, genetically determined dementia, or disorders excluded from the study.
4. Participants unwilling to participate in the study.
5. The presence of a physical ailment that is incapacitating or hinders communication (Grzenda et al., 2024).

### Yoga intervention

The yoga-based intervention consisted of daily 60-minute sessions, 6 days a week, for 12 weeks, conducted by a trained yoga therapist in the Yoga Room of the Department of Anatomy at AIIMS, New Delhi. Patients were also instructed to practice yoga at home and maintain a yoga diary throughout the 12-week period. Each session included 10 min of Surya Namaskara, which comprised 12 steps; 5–7 min of deep relaxation through the Muscle Relaxation Technique; and 15 min of asanas (yoga postures), including Pavanamuktasana (supine position), Bhujangasana and Shalabhasana (prone position), Ardhamatsyaendrasana and Mandukasan (sitting position), followed by standing positions such as Tadasana, Trikonasana, and Ardhachakrasana, as recommended for diabetes management by yoga experts. This was followed by 15 min of pranayama, including Kapalbhati, Bhastrika, Nadi Shodhan, and Bhramari. Each session concluded with 5–10 min of meditation. A record of the patients' diet, medications, and exercise details was maintained throughout the study period.

### Neurocognitive assessment for quality of life (QOL) in AD subjects versus healthy control (baseline)

Socio-demographic and lifestyle data, including sex, age, education, employment, family status, and housing arrangements, were collected. Using the following assessments, an experienced neuropsychologist evaluated each patient’s mood and overall cognitive functioning:

### Geriatric depression scale (GDS)

The GDS was used to assess the QOL of AD subjects before and after the yoga intervention. The GDS includes 15 questions, with scores indicating the presence of depression based on answers provided by the AD subjects (Joe et al., 2024). Of the 15 questions, 10 (questions 1, 5, 7, 11, and 13) identify depression when answered affirmatively, while the remaining questions indicate depression when answered negatively. Depending on age, education, and complaints, the scoring is as follows: 0–4 is considered normal, 5–8 indicates mild depression, 9–11 denotes moderate depression, and 12–15 suggests severe depression. The GDS Short Form is more effective for individuals with physical illness or mild to moderate AD who may have short attention spans or become easily fatigued. This version takes 5–7 min to complete. Compared to diagnostic criteria, the GDS demonstrated 89% specificity and 92% sensitivity, and its validity and reliability have been validated through research and clinical experience (Öksüz et al., 2024).

### Montreal cognitive assessment (MoCA)

The total MoCA score (out of 30) was assessed before and after the yoga intervention. The individual scores for specific cognitive domains such as language, memory, attention, visuospatial skills, naming, delayed recall, abstraction, and orientation were also recorded. The MoCA is a 30-point, one-page test that takes approximately 10 min to complete. It evaluates orientation (6 points), clock-drawing ability (3 points), cube copying (1 point), and short-term memory (5 points). Modified Trail Making Part B (1 point), phonemic fluency (1 point), and verbal abstraction (2 points) are used to assess executive function. Working memory, attention, and focus are tested through a sustained-attention task (1 point), digit span (2 points), and serial calculations (3 points) (Furneri et al., 2024). Additionally, sentence repetition (2 points), fluency tasks, and naming obscure species (3 points) assess linguistic proficiency. For patients with fewer than 12 years of education, the assessor adds 1 point to their score. With a cut-off score of 26, item analysis has shown that the MoCA can reliably distinguish between patients before and after the intervention.

### Caregiver burden assessment

The Caregiver Burden Scale (CB Scale) is used to assess the subjective burden experienced by caregivers. The questionnaire consists of 22 questions, which are categorized into five dimensions: general strain (questions 1, 3, 4, 5, 7, 10, 14, 19), the environment (questions 9, 15, 17), social isolation (questions 8, 12, 22), emotional involvement (questions 6, 11, 16), and disappointment (questions 2, 13, 18, 20, 21). Each question has four possible responses, with scores ranging from one to four points. The final score is the average of the scores from all five dimensions. Based on the overall score, CB can be categorized into three levels: low (1.00–1.99 points), medium (2.00–2.99 points), and high (3.00–3.99 points) (Kowalska et al., 2017).

### Quality of life (QOL)

QOL is a complex, subjective experience shaped by various factors, including time, social status, culture, and physical health. While there is no universally accepted definition, researchers agree on its multifaceted nature. QOL is often described as: (a) multidimensional, reflecting the fact that life includes multiple dimensions such as social, mental, material, physical, cultural, and economic aspects; (b) dynamic, due to its evolving nature across space and time; and (c) subjective, as it is influenced by individual perceptions and the personal significance attributed to experiences both within and between individuals (Aye et al., 2023). QOL scores were derived from the GDS and MoCA assessments post-intervention.

### Data analysis

For statistical analysis, all collected data were entered into a database using the Windows version 10.0 of the Statistical Package for the Social Sciences (SPSS). Descriptive statistics were used to summarize participants' profiles and QOL. A Student’s t-test was applied to compare overall QOL scores between groups. The means ± standard deviation (SD) was used to describe quantitative variables, while percentages and counts were used for qualitative data. Correlational analyses (Pearson’s r) were conducted to explore the relationships between CI, mood, behavioral symptoms, functional capacities, and QOL.

## Results

### Demographics and characteristics outcome

AD was found to be inversely correlated with quality of life, with patient demographics and characteristics playing a significant role. The relationship between QOL and AD showed a notable improvement following 12 weeks of yoga practice (P < 0.01). Baseline demographics were assessed at time point T0, i.e. baseline (pre-yoga), and correlations between characteristics were evaluated by comparing assessments at T12 (post-yoga), as shown in (Table 1). Regarding social activity status, pre-yoga data showed 6.67% of participants were non-social, while 93.33% were socially active. Post-yoga, the percentage of non-social participants decreased to 4.80%, and socially active participants increased to 95.20%. In terms of health status, pre-yoga data revealed 40.00% of participants reported stress, 26.67% reported depression, and 33.33% reported hypertension. Post-yoga, these scores decreased to 28.50% for stress, 17.00% for depression, and 21.90% for hypertension. Both social activity and health status showed significant improvement following the yoga intervention, as presented in (Table 2).

**TABLE 1:** Demographics and clinical characteristics of AD participants.

Demographics and clinical characteristics (AD participants)	Total (N = 30), mean ± SD or number or percentage
Age, years	66.4 ± 3.80
Gender	
Female	40.00%
Male	60.00%
Height	165.53 ± 9.83
Weight	66.73 ± 8.86
Education	
_10_th	23.33%
_12_th	43.33%
Graduate	33.33%
Occupation	
Business	30.00%
Government	30.00%
Army	3.33%
Corporate	13.33%
Driver	3.33%
Housewife	13.33%
Police	3.33%
Truck driver	3.33%
Marital status	
Married	96.67%
Unmarried	3.33%
Social status	
Not social	6.67%
Socialite	93.33%
Economic status	100%
Health Status	
Stress	40.00%
Depression	26.67%
Hypertension	33.33%
Alcohol	
No	43.33%
Yes	56.67%
Smoking	
No	46.67%
Yes	53.33%
Diet	
NonVeg	23.33%
Veg	76.67%
Family history	
No	80.00%
Yes	20.00%
Past medical history	
CVS	13.33%
Diabetes	6.67%
Hypertension	3.33%
Stroke	3.33%
No	43.33%
Age of onset	63.73 ± 2.49
Age of clinical diagnosis (year)	2023
Neuropsychological assessment scales	
GDS	8.36 ± 2.79
MoCA	18.23 ± 4.90
Caregiver	
No	86.67%
Yes	6.67%

Data represents demographics and clinical characteristics of Alzheimer’s Disease (AD) participants (N = 30). Percentages are calculated based on the total number of participants. Age, height, and weight values are reported as mean ± standard deviation. Health status includes conditions such as stress, depression, and hypertension. The family history and past medical history details reflect the presence or absence of related conditions. Neuropsychological assessments are based on the Geriatric Depression Scale (GDS) and Montreal Cognitive Assessment (MoCA). The caregiver information indicates whether participants had a caregiver.

**TABLE 2:** Demographic and clinical characteristics of AD patients at pre- and post-Yoga intervention.

Characteristics (patients)	Total (N = 30) pre- yoga, mean ± SD percentage	Total (N = 30) Post-yoga, mean ± SD or percentage
Social status		
Not social	6.67%	4.80%
Socialite	93.33%	95.2%
Health status		
Stress	40.00%	28.50%
Depression	26.67%	17.00%
Hypertension	33.33%	21.90%

Values represent mean ± SD (standard deviation) for continuous variables and percentage (%) for categorical variables. Health status percentages represent the prevalence of conditions reported by participants at baseline (pre-yoga) and post-intervention (post-yoga).

#### Yoga reduces GDS

**FIGURE 2:**
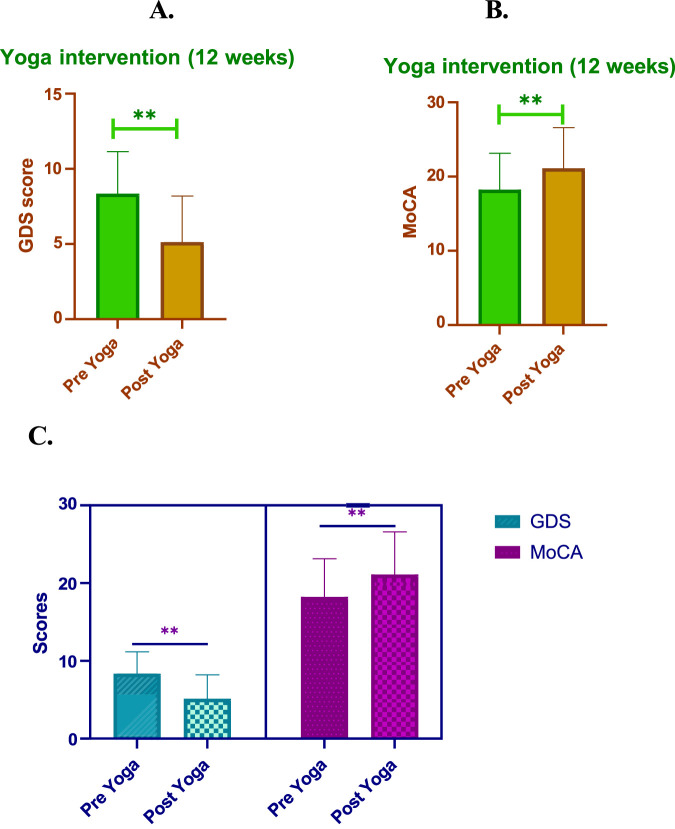
Impact of 12-weeks of post-yoga intervention in GDS and MoCA scores **(A)**. Yoga intervention (12 weeks). **(B)** Yoga intervention (12 weeks). This figure illustrates the changes observed in Geriatric Depression Scale (GDS) and Montreal Cognitive Assessment (MoCA) scores following a 12-week yoga intervention in individuals with mild to moderate AD. **(A)** GDS Scores: Significant reduction in depression symptoms post-intervention, with GDS scores decreasing from 8.36 ± 2.79 (pre-yoga) to 5.13 ± 3.07 (post-yoga), indicating enhanced mood and quality of life. **(B)** Total MoCA Scores: Overall cognitive function, as measured by MoCA, showed improvement from 18.23 ± 4.90 to 21.1 ± 5.09, highlighting enhanced cognitive abilities post-yoga intervention. **(C)** MoCA Component Scores: Individual domains within the MoCA, including language, attention, visuospatial ability, naming, delayed recall, abstraction, and orientation, also exhibited noticeable improvements post-intervention, reflecting the holistic impact of yoga on cognitive performance.

The GDS demonstrated a significant decrease from pre-yoga (8.36 ± 2.79) to post-yoga (5.13 ± 3.07), with the change being statistically significant (P < 0.01). The 15 questions assessed are presented in Table 3 and scores were shown in Figure 2A. Additionally, the change in QOL, measured as 3.23 ± 1.59 in the post-yoga neurocognitive assessments, also showed a statistically significant improvement (P < 0.01) in AD patients. These findings suggest that yoga can be an effective intervention to reduce depression, improve cognitive function, and enhance social interactions in AD patients, making it a valuable adjunct to traditional therapies.

**TABLE 3:** Effect of Yoga on cognitive function and depression in AD patients (pre- and post-12 weeks Yoga intervention).

Scale	Pre-yoga	Post-yoga (12 weeks)	*P value*
GDS	8.36 ± 2.79	5.13 ± 3.07	*P < 0.01*
MoCA	18.23 ± 4.90	21.1 ± 5.49	*P < 0.01*
Language	1.00 ± 0.2	2.00 ± 0.6	*P < 0.01*
Memory	no points	No points	*----------*
Attention	3.02 ± 1.7	4.00 ± 1.5	*P < 0.01*
Visuospatial	4.00 ± 1.3	4.00 ± 1.50	*P < 0.01*
Naming	2.06 ± 0.4	2.00 ± 0.74	*P < 0.01*
Delayed Recall	3.05 ± 1.2	4.00 ± 0.50	*P < 0.01*
Abstraction	1.10 ± 0.1	1.50 ± 0.20	*P < 0.01*
Orientation	3.00 ± 1.1	4.00 ± 0.45	*P < 0.01*

Values represent mean ± standard deviation. GDS, geriatric depression scale; MoCA, Montreal Cognitive Assessment. Statistical significance indicated by P < 0.01 for all outcomes pre- and post-yoga intervention.

#### Yoga improves MoCA scores

The individual MoCA domain scores demonstrated significant improvements post-yoga intervention, highlighting the cognitive benefits across various cognitive functions in AD patients. Notably, language skills showed an increase from 1.00 ± 0.2 pre-yoga to 2.00 ± 0.6 post-yoga, indicating a notable improvement in verbal fluency. Attention, which improved from 3.02 ± 1.7 to 4.00 ± 1.5, suggests enhanced focus and concentration abilities. The visuospatial domain remained stable, with scores of 4.00 ± 1.3 pre-yoga and 4.00 ± 1.5 post-yoga, but still reflects the consistency of spatial awareness. Memory showed no significant change, with scores remaining constant, while delayed recall demonstrated a meaningful improvement, increasing from 3.05 ± 1.20 to 4.00 ± 0.50. This indicates a strengthening of the ability to retain and recall information after a delay. Abstraction, a measure of executive function, decreased slightly from 1.10 ± 0.1 to 1.50 ± 0.20, which reflect a shift in cognitive improvement. Lastly, orientation saw a significant improvement, with scores increasing from 3.00 ± 1.1 to 4.00 ± 0.45, suggesting enhanced awareness of time and place. Overall, these domain-specific improvements contribute to the observed overall increase in MoCA scores (P < 0.01), further emphasizing the cognitive benefits of yoga in AD patients. These domain-specific improvements underscore the significance of yoga as a comprehensive intervention for enhancing cognitive function in multiple areas, improving overall quality of life (Table 4; Figure 2B).

**TABLE 4:** Effect of 12-week Yoga intervention on GDS and MoCA scores and their Impact on quality of life.

Yoga intervention (12 weeks)	GDS Score	Change in QOL (GDS Score)	*P value*	MoCA Score	Change in QOL (MoCA Score)	*P value*
Pre-yoga	8.36 ± 2.79	3.23 ± 1.59	*P < 0.01*	18.23 ± 4.90	2.86 ± 2.70	*P < 0.01*
Post-yoga	5.13 ± 3.07			21.1 ± 5.49		

GDS, Score: Geriatric Depression Scale; MoCA, Score: Montreal Cognitive Assessment; QOL, Quality of Life. P-values are derived from paired t-tests comparing pre- and post-yoga intervention scores. Changes in GDS score represented as pre-yoga minus post yoga intervention and changes in MoCA score represented as post- yoga minus pre-yoga.

#### Yoga improves overall quality of life (QOL)

The 12-week yoga intervention resulted in significant improvements in both quality of life (QOL) and neurocognitive function in AD patients. As measured by the GDS, the pre-yoga QOL score was 8.36 ± 2.79, which significantly decreased to 5.13 ± 3.07 post-yoga (P < 0.01). Similarly, the change in QOL measured by the GDS score showed a notable improvement of 3.23 ± 1.59 (P < 0.01). In terms of cognitive function, the MoCA score increased from 18.23 ± 4.90 pre-yoga to 21.1 ± 5.49 post-yoga, demonstrating a statistically significant improvement (P < 0.01). The change in QOL based on MoCA was 2.86 ± 2.70 (P < 0.01), reflecting a positive impact on cognitive function following yoga intervention. These findings highlight the significant potential of yoga in improving the QOL and cognitive function of AD patients, suggesting it as an effective adjunctive therapy for enhancing mental and cognitive wellbeing in this population. Figure 2C, Table 4.

#### Yoga reduces to caregiver burden (CB)

The pre-yoga CB scale revealed that 6.67% of caregivers reported a high burden, while 86.67% of caregivers reported a medium degree of burden (Table 1). Among the subscales, the “environment” subscale showed the lowest burden (1.7 ± 0.5 points), while the “general strain” subscale exhibited the highest burden (2.9 ± 0.6 points). Post-yoga, the CB Scale demonstrated a significant reduction in CB. The “environment” subscale burden decreased to 1.4 ± 0.2 points, while the “general strain” subscale showed a reduction to 2.1 ± 0.3 points, reflecting an overall decrease in CB. These changes indicate a shift towards a lower degree of load, suggesting that yoga intervention led to a significant reduction in the strain experienced by caregivers of AD patients (Table 5).

**TABLE 5:** Caregiver burden scores at pre- and 12-weeks of pot-Yoga intervention.

Caregiver burden (mean ± SD)	Environ ment	Emotional involvement	Disappointment	Social isolation	General strain
Pre-yoga	1.7 ± 0.5	2.0 ± 1.5	2.5 ± 0.1	2.2 ± 0.3	2.9 ± 0.6
Caregiver burden (mean ± SD) Post-Yoga	1.4 + 0.2	1.7 ± 0.8	1.9 ± 0.5	2.0 ± 0.1	2.1 ± 0.3

Values represent the mean ± SD (standard deviation) of caregiver burden scores in various domains before and after a 12-week yoga intervention. The domains assessed include Environment, Emotional Involvement, Disappointment, Social Isolation, and General Strain.

The reduction in CB underscores the positive impact of yoga on both AD patients and their caregivers.

## Discussion

Cognitive impairment (CI), including MCI and AD, has been steadily rising as the global population ages, significantly affecting QOL and health span (Gholamalishahi et al., 2023); (Madhuri et al., 2017). MCI, often considered the prodromal stage of AD, is characterized by measurable cognitive decline without a loss of functional independence (Zhang et al., 2024). Symptoms such as anxiety, irritability, and depression are common in MCI and are associated with a higher risk of progression to AD (Cox et al., 2024). Current evidence suggest that 5%–15% of MCI cases advance to AD annually, underscoring the critical need for effective interventions (Karamacoska et al., 2023). Furthermore, research indicates that individuals with MCI are more susceptible to anxiety and depressive disorders, which are prevalent among older adults with MCI (Jain et al., 2023). Additionally, research has demonstrated that anxiety and depression are risk factors for MCI and that AD frequently develops before these disorders (Lanctôt et al., 2024a). Our study demonstrates that a 12- week yoga intervention significantly improved the QOL and neurocognitive function in AD patients. Baseline assessments (T0) highlighted the detrimental effects of AD on QOL, with significant improvements observed at post-intervention (T12). Notably, there was a marked reduction in depressive symptoms (GDS scores decreased from 8.36 ± 2.79 to 5.13 ± 3.07, *P* < 0.01) and a corresponding enhancement in QOL, as measured by neurocognitive assessments (change in QOL score: 3.23 ± 1.59, *P* < 0.01). These findings align with previous studies advocating for non-pharmacological, holistic interventions like yoga to manage cognitive and emotional symptoms in AD (Brown and Bayley, 2024; Raza et al., 2024). Cognitive function, assessed using the MoCA showed significant domain-specific improvements post-yoga intervention. Language scores improved from 1.00 ± 0.2 to 2.00 ± 0.6, while attention scores increased from 3.02 ± 1.7 to 4.00 ± 1.5 (*P* < 0.01). Although memory scores remained stable, delayed recall exhibited a meaningful enhancement (from 3.05 ± 1.20 to 4.00 ± 0.50), indicative of better information retention. Orientation and visuospatial abilities also improved, emphasizing yoga’s comprehensive cognitive benefits (Table 4; Figure 2C). These findings corroborate evidence suggesting that yoga enhances neuroplasticity and promotes cognitive resilience in aging populations (Lanctôt et al., 2024b; Voss et al., 2023). Beyond cognitive improvements, yoga also positively influenced participants' social activity and health status. Pre-intervention, 40.00% of participants reported stress, 26.67% reported depression, and 33.33% reported hypertension. Post-yoga, these figures declined to 28.50%, 17.00%, and 21.90%, respectively, alongside an increase in social engagement. Such outcomes emphasize yoga’s role in improving mental wellbeing, reducing psychological distress, and fostering social connectedness, all of which are critical for maintaining QOL in AD patients (Table 2). CB, often overlooked in AD management, showed significant reductions post-yoga. The “general strain” subscale decreased from 2.9 ± 0.6 to 2.1 ± 0.3, reflecting reduced stress among caregivers. This suggests that yoga not only benefits AD patients but also alleviates caregiver strain, highlighting its dual impact on patient-caregiver dynamics (Table 5). Our findings align with prior research indicating that physical activity, including yoga, can enhance cognitive function and mental health in older adults (Eilat-Adar et al., 2023; Vejandla et al., 2024).

The benefits observed in this study may be attributed to yoga’s multifaceted effects, including stress reduction, improved autonomic regulation, and neuroendocrine modulation. Additionally, yoga’s emphasis on mindfulness and breathing exercises likely contributes to enhanced emotional regulation and cognitive focus. Recent studies from our lab have documented that yoga based lifestyle intervention can reduce biological age and prolong health span with lifespan (Tolahunase et al., 2018a; Gautam et al., 2021; Baljinder et al., 2024). Yoga has been shown to improve both mitochondrial and nuclear genomic integrity. Telomeres are influenced by several factors, but two primary determinants are free radical levels and the activity of the telomerase enzyme (Madhuri et al., 2017). Improvements in mitochondrial health led to a reduction in oxidative stress, enhanced ATP production, and help prevent the accelerated attrition of telomeres and yoga also increases the activity and levels of Telomerase reverse transcriptase which aids in maintenance of telomere length and thus reduces our biological age. High levels of stress, anxiety and depression are associated with accelerated biological aging, high levels of inflammation and cortisol. Yoga decreases inflammation, reduces cortisol levels induces relaxation response and reduces inflammation associated oxidative stress (Tolahunase et al., 2018b). All these factors are also predisposing factors for early cognitive decline in AD. Yoga also increases melatonin key molecule to maintain sleep wake cycle and thus yoga aids in maintain restful and restorative sleep and can reduce inflammation, modulate immune response and help in memory potentiation (Dada et al., 2022). Current study proves that yoga intervention can improve the lifestyle and help in the prevention of AD. Yoga influences several biological pathways, contributing to its therapeutic benefits. One key pathway is Brain-Derived Neurotrophic Factor (BDNF) regulation, where yoga has been shown to elevate BDNF levels, promoting neuroplasticity, cognitive function, and stress resilience (Naveen et al., 2013). Tau modulation is another critical area, as yoga may reduce hyperphosphorylation of tau proteins, potentially mitigating neurodegenerative processes associated with AD (Clemente-Suárez et al., 2024a) Additionally, yoga impacts inflammatory pathways by downregulating pro-inflammatory markers such as IL-6, TNF-α, and CRP, while upregulating anti-inflammatory cytokines like IL-10, thereby fostering an anti-inflammatory state (Vijayaraghava et al., 2015). Together, these pathways underline yoga’s role in enhancing mental health, reducing neurodegeneration, and alleviating systemic inflammation. There are currently limited pharmacological treatments available for MCI (Ahmad et al., 2024), which highlights the necessity for efficient non-pharmacological therapies. Mindfulness-based therapies, including yoga, have shown potential in improving various health factors, such as depression, stress, cognitive decline, immune system changes, and brain structural alterations. These factors are associated with an increased risk of progression from MCI to dementia (Gautam et al., 2019). Despite multiple clinical trials, the FDA has yet to approve any effective medications for treating MCI or delaying the onset of AD. In this context, non-pharmacological therapies, particularly those based on holistic mind-body practices like yoga, have emerged as promising complementary interventions. Yoga has shown potential in improving cognitive function, reducing stress, and enhancing overall wellbeing in individuals with MCI and AD (Brenes et al., 2019; Clemente-Suárez et al., 2024b). However, it is important to acknowledge that while yoga offers significant benefits, it should be viewed as an adjunct to pharmacological treatments, rather than a substitute, and further research is necessary to fully understand its role in managing these conditions. Yoga may prevent or delay the onset of AD as well as the risk of cognitive decline and death, according to the majority of longitudinal epidemiological research, which has decisively shown dose-response connections between physical activity and the risk of cognitive decline (Brenes et al., 2019). Physical exercise was associated with a decreased risk of dementia from all causes, AD, and vascular dementia, even in long-term follow-ups for AD and all-cause dementia. A significant decrease in AD cases in Italy could be achieved with primary prevention (Deng et al., 2024). Despite the promising results observed in this study, several limitations must be considered. The research was conducted in a single-center, hospital-based setting with a relatively small sample size, which may limit the generalizability of the findings. Future studies should aim to explore the long-term benefits of yoga, particularly through longitudinal research, to assess its sustained impact on different AD phenotypes, including non-amnestic variants. Investigating the neurobiological mechanisms underlying yoga’s effects, such as its influence on tau and amyloid pathology, could provide valuable insights into its therapeutic potential.

Additionally, larger and more diverse cohorts are needed to validate the efficacy of yoga-based interventions across a broader population (Dolphin et al., 2024). Our study found a strong positive correlation between QOL social ties, psychological health, and the living environment in patients with low to moderate AD. Factors like age, chronic illnesses, and body mass index (BMI) were directly correlated with AD, while BMI and QOL were negatively impacted by AD, though yoga was found to significantly reduce BMI and inflammatory cytokine levels (Chen et al., 2020). These findings support the potential of yoga-based interventions to enhance both cognitive and physical wellbeing in senior MCI patients in India. The results emphasize the importance of developing customized yoga therapies to improve the QOL, executive function, and overall wellbeing of AD patients and their caregivers.

## Conclusion

Yoga, as a mind-body energy medicine, is a promising non-pharmacological holistic approach with potential for management, prevention, and rehabilitation in MCI and AD. Our findings demonstrated significant improvements in QOL and cognitive function in AD patients, with reductions in depressive symptoms, enhanced cognitive performance, and increased social engagement. Additionally, the intervention alleviated CB, benefiting not only the patients but also their caregivers. These results suggest that yoga may serve as a valuable complement to traditional treatments, promoting both mental wellbeing and overall QOL. Future studies should address limitations such as the short intervention duration, small sample size, and lack of long-term follow-up. Research should focus on understanding the underlying mechanisms, exploring the effects of different yoga modalities, and assessing its benefits across various stages of MCI and AD. Larger, multi-center trials are necessary to validate these findings and evaluate the sustained effectiveness of yoga as a therapeutic intervention in dementia care.

## Data Availability

The raw data supporting the conclusion of this article will be made available by the authors, without undue reservation.
